# Mechanical diversity and folding intermediates of parallel-stranded G-quadruplexes with a bulge

**DOI:** 10.1093/nar/gkab531

**Published:** 2021-06-17

**Authors:** Yashuo Zhang, Yuanlei Cheng, Juannan Chen, Kewei Zheng, Huijuan You

**Affiliations:** Hubei Key Laboratory of Natural Medicinal Chemistry and Resource Evaluation, School of Pharmacy, Tongji Medical College, Huazhong University of Science and Technology, Wuhan 430030, China; Hubei Key Laboratory of Natural Medicinal Chemistry and Resource Evaluation, School of Pharmacy, Tongji Medical College, Huazhong University of Science and Technology, Wuhan 430030, China; School of Pharmaceutical Sciences (Shenzhen), Sun Yat-Sen University, Guangzhou 510275, China; School of Pharmaceutical Sciences (Shenzhen), Sun Yat-Sen University, Guangzhou 510275, China; Hubei Key Laboratory of Natural Medicinal Chemistry and Resource Evaluation, School of Pharmacy, Tongji Medical College, Huazhong University of Science and Technology, Wuhan 430030, China

## Abstract

A significant number of sequences in the human genome form noncanonical G-quadruplexes (G4s) with bulges or a guanine vacancy. Here, we systematically characterized the mechanical stability of parallel-stranded G4s with a one to seven nucleotides bulge at various positions. Our results show that G4-forming sequences with a bulge form multiple conformations, including fully-folded G4 with high mechanical stability (unfolding forces > 40 pN), partially-folded intermediates (unfolding forces < 40 pN). The folding probability and folded populations strongly depend on the positions and lengths of the bulge. By combining a single-molecule unfolding assay, dimethyl sulfate (DMS) footprinting, and a guanine-peptide conjugate that selectively stabilizes guanine-vacancy-bearing G-quadruplexes (GVBQs), we identified that GVBQs are the major intermediates of G4s with a bulge near the 5′ or 3′ ends. The existence of multiple structures may induce different regulatory functions in many biological processes. This study also demonstrates a new strategy for selectively stabilizing the intermediates of bulged G4s to modulate their functions.

## INTRODUCTION

G-quadruplexes (G4s) are four-stranded nucleic acid structures formed by Hoogsteen base pairing of guanines and further stabilized by monovalent cations, such as K^+^ or Na^+^ (1–6). Bioinformatic analyses performed using the consensus sequence formula G_≥3_N_1–7_G_≥3_N_1–7_G_≥3_N_1–7_G_≥3_ revealed more than 300,000 putative G4-forming sequences in the human genome, where G refers to adjacent guanines and N refers to bases in the loop region (7,8). Recently, the definition of G4 structures has been broadened by structural studies of bulged G4s (9,10), long-looped G4s (11), guanine-vacancy-bearing G4s (GVBQs) (12–15), and two-tetrad G4s (16). High-throughput chip-sequencing experiments detected more than 700,000 potential G4-forming sequences in the human genome (17). Among them, ∼70% of the total observed G4-forming sequences are noncanonical G4s, including bulged G4s (∼30%), long looped G4s (∼24%), GVBQs, two-tetrad G4s, and G4s comprising both long loops and bulges (∼14%) (17).

Bulged G4s have been observed in the functional regions of the human genome, such as the human telomerase RNA component (hTERC) (18), KRAS proto-oncogene promoter (19), and poly (ADP-ribose) polymerase-1 (PARP1) promoter (20). Bulged G4-forming sequences have also been identified in the proviral HIV-1 genome (21) and used as the HIV-1 integrase inhibitor T30177 (9). A luciferase activity experiment suggested that bulged G4s formation correlates with HIV-1 LTR promoter activity in HEK 293T cells (21). GVBQs are also important noncanonical G4 structures observed in the human genome (12,15,22). Recent studies have suggested that GVBQs can bind and be stabilized by physiologically relevant guanine metabolites (GMPs), secondary messengers (cyclic dinucleotides), guanine-derivative drugs (e.g. acyclovir), and guanine-peptide conjugates, thereby serving as novel drug targets (12,15,23,24).

NMR structural studies showed that the HIV-1 integrase inhibitor T30177 formed a parallel-stranded G4 structure with one nucleotide (nt) thymine bulge that connects two adjacent guanines of one column (9). All 12 guanines in this sequence participated in G-tetrad formation. A systematic analysis revealed that the bulge can form in 8 different positions between the 8 successive guanine pairs with sizes of 1 to 7 nt, and all these sequences form parallel-stranded G4s (10). Circular dichroism (CD) and NMR melting curve analysis revealed that the bulge reduced the thermodynamic stability of G4s compared with the original T30695 sequence, which does not have a bulge. The stability of the bulged G4s depends on the size and the position of the bulges, in which the TB-1 sequence (Table 1) has the highest melting temperature (*T*_m_) compared to other sequences (TB-2 to TB-8) (10). When the bulge length increased from 1 nt (TB-1) to 7 nt (T7B-1), *T*_m_ decreased >37°C in 60 mM K^+^ buffer (10). However, G4-forming sequences often exhibit complex folding energy landscapes and diverse folding conformations (i.e., long-lived intermediates) (25,26). These ensemble methods encounter difficulty in resolving the stability of multiple coexisting G4 structures.

**Table 1.** G4-forming sequences with a bulge used in this study

Name	Sequence (5′ to 3′)			
T30695	TTGGGT	GGGT	GGGT	GGGT
TB-1	TTGTGGT	GGGT	GGGT	GGGT
TB-2	TTGGTGT	GGGT	GGGT	GGGT
TB-3	TTGGGT	GTGGT	GGGT	GGGT
TB-4	TTGGGT	GGTGT	GGGT	GGGT
TB-5	TTGGGT	GGGT	GTGGT	GGGT
TB-6	TTGGGT	GGGT	GGTGT	GGGT
TB-7	TTGGGT	GGGT	GGGT	GTGGT
TB-8	TTGGGT	GGGT	GGGT	GGTGT
T3B-1	TTGTTTGGT	GGGT	GGGT	GGGT
T5B-1	TTGTTTTTGGT	GGGT	GGGT	GGGT
T7B-1	TTGTTTTTTTGGT	GGGT	GGGT	GGGT
T3B-8	TTGGGT	GGGT	GGGT	GGTTTGT
T5B-8	TTGGGT	GGGT	GGGT	GGTTTTTGT
T7B-8	TTGGGT	GGGT	GGGT	GGTTTTTTTGT
T2B-2	TTGGTTGT	GGGT	GGGT	GGGT
T3B-2	TTGGTTTGT	GGGT	GGGT	GGGT
T5B-2	TTGGTTTTTGT	GGGT	GGGT	GGGT
T7B-2	TTGGTTTTTTTGT	GGGT	GGGT	GGGT
T2B-3	TTGGGT	GTTGGT	GGGT	GGGT
T3B-3	TTGGGT	GTTTGGT	GGGT	GGGT
T5B-3	TTGGGT	GTTTTTGGT	GGGT	GGGT
T7B-3	TTGGGT	GTTTTTTTGGT	GGGT	GGGT
T2B-4	TTGGGT	GGTTGT	GGGT	GGGT
T2B-5	TTGGGT	GGGT	GTTGGT	GGGT
T2B-6	TTGGGT	GGGT	GGTTGT	GGGT
T2B-7	TTGGGT	GGGT	GGGT	GTTGGT

Single-molecule force spectroscopy techniques have become useful tools for studying the mechanical diversity of G4 structures (27–37). The Mao lab reported that the ILPR promoter sequence forms two different G4 conformations with distinct mechanical stability (28). Based on the unfolding force distributions and unfolding step size distributions, the Mao lab identified multiple intermediates of hTERT promoter G4s (29) and human telomeric G4s (30). By combining optical tweezers and fluorescence resonance energy transfer (FRET), the Ha lab observed that telomere G4-forming sequences can form more than six stable secondary structures with distinct mechanical stability (34,35). Hence, single-molecule measurements of mechanical stability are well suited for studies seeking to characterize the polymorphism of G4 formation. In addition, DNA G4s are subjected to various mechanical modifications during transcription and replication. DNA or RNA polymerases and helicases function as motor proteins tracking along with DNA and exerting forces on G4 structures (38). Therefore, the measurements of the mechanical stability of G4s provide insights for understanding the interactions between G4s and the motor proteins (39). However, the mechanical stability and conformational diversity of noncanonical G4s remain poorly understood compared with canonical G4s.

Herein, we systematically investigated the mechanical stability of noncanonical parallel-stranded G4s containing a one to seven nt bulge at different positions (Table 1) and 12 GVBQs (Supplementary Table S1) by using single-molecule magnetic tweezers. To identify the potent intermediates of G4s with a bulge located near the 5′ or 3′ end, we used a guanine-RHAU23 peptide conjugate (GRPC), which selectively stabilizes the GVBQs (24). This bifunctional GRPC was composed of a guanine base and a G4 binding domain (RHAU23) from the RHAU helicase (24).

## MATERIALS AND METHODS

### Oligonucleotides, peptide, DNA sample preparation

Oligonucleotides were purchased from Sangon Biotech Co., Ltd (China) and Genewiz, Inc (China). GRPC peptide (a guanine moiety connect to a 23 amino acids peptide HPGHLKGREIGMWYAKKQGQKNK) was purchased from SBS Genentech (China) as previously described (24). The DNA constructs containing the G4-forming sequence for single-molecule experiments were prepared as previously described (40,41).

### CD spectroscopy

The CD spectra and CD melting curves were collected on a Jasco-810 spectropolarimeter using a 1-mm optical path length quartz cuvette. Scans were performed in a range of 220 to 320 nm for five times at room temperature with a speed of 500 nm/min. The oligonucleotides were dissolved in 10 mM Tris–HCl (pH 8.0) buffer containing 100 mM and 20 mM KCl for CD spectra and CD melting curve measurements, respectively. The oligonucleotides were heated at 95°C for 5 min and slowly cooled to room temperature. The melting experiments were performed at several temperature points during heating from 20°C to 85°C (1.0°C/min) and the temperature was equilibrated for 5 min before recording. The melting curve profile was fitted by a sigmoid function utilizing the absorbance at 265 nm.

### Dimethyl sulfate (DMS) footprinting

Fluorescein (5′-FAM) labeled oligonucleotides (0.1 μM) dissolved in 10 mM Tris–HCl (pH 8.0) and 0.5 μM EDTA buffer were heated at 95°C for 5 min and subsequently cooled to room temperature. Next, 100 mM LiCl, 100 mM KCl or 0.5 μM GRPC/100 mM KCl was added to the oligonucleotides, and the mixtures were immediately treated with 5% DMS reagent (Innochem, China) for 2 min on ice. Reactions were terminated by adding 80 μl stop buffer (3 M sodium acetate, 0.1 M β-mercaptoethanol, and 1 mg/ml spermidine DNA). After chloroform extraction and ethanol precipitation, DNA was cleaved by the addition of 10%, vol/vol piperidine (Sinopharm Chemical Reagent Co.,Ltd, China) and incubated at 90°C for 30 min. DNA was purified again by chloroform extraction and ethanol precipitation and dissolved in 80% (vol/vol) deionized formamide (Sangon Biotech Co., Ltd, China). The DNA samples were boiled at 95°C for 5 min and subsequently cooled on ice for 15 min before being loaded onto a 20% denaturing polyacrylamide gel. DNA fragments were visualized by iBright 1500 (Thermo Fisher Scientific, USA) and digitized by ImageQuant 5.2 software.

### Magnetic tweezers experiments

Vertical magnetic tweezers (BioPSI, Singapore) were used in this work (42). A flow chamber was built on a (3-aminopropyl) triethoxy silane (APTES, Cool Chemical Technology, China) modified coverslip. The APTES coverslip was reacted with Sulfo-SMCC (Thermo Fisher Scientific, USA) first and then with 5′-thiol-labeled DNA to covalently link the G4 DNA constructs on the coverslip (32). The chamber was blocked with 10 mg/ml bovine serum albumin (BSA) (Sigma-Aldrich, China), and 1 mM 2-mercaptoethanol in 1× phosphate-buffered saline (PBS) buffer (pH 7.4) for more than 2 hours. Streptavidin-coated paramagnetic beads (Dynal M280, Thermo Fisher Scientific, USA) were introduced to attach the 5′-biotin end of the DNA constructs. All data were collected at room temperature (23-25°C) in assay buffer (10 mM Tris–HCl, 100 mM KCl, and pH 8.0). The unfolding forces were measured using the force-ramp procedure at a 2 pN/s loading rate from 1 to 60 pN and subsequently jumped to 1 pN for more than 30 s for G4 refolding. The unfolding force distributions of each sequence were obtained from more than 100 repeating force-ramp cycles and more than five independent DNA molecules. The unfolding force distributions were analyzed by Bell's model (Supplementary Data analysis). The unfolding step sizes were analyzed using a home-built MATLAB program, as previously described (32,43).

## RESULTS

### Mechanical stability of TB-1 to TB-8 G4s bearing a 1 nt bulge depends on bulge positions

**Figure 1.**
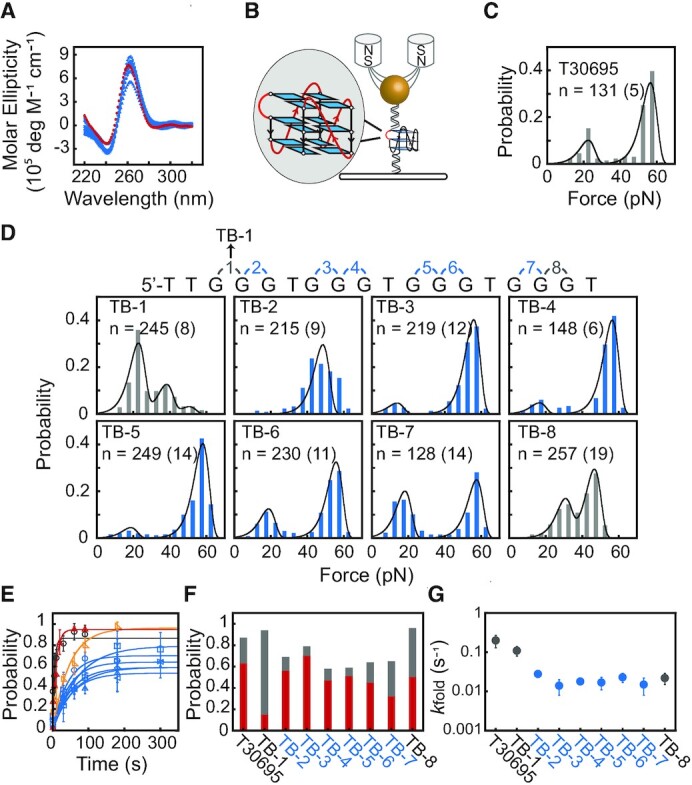
Mechanical stability of G4s bearing a 1 nt bulge. (**A**) CD spectra of T30695 (red) and TB-1 to TB-8 DNA (cyan). (**B**) Schematic of the magnetic tweezers experiments. The G4-forming sequence is sandwiched between two dsDNA handles. (**C**) Unfolding force distribution of T30695. (**D**) Unfolding force distributions of TB-1 to TB-8. Data were fitted by Bell's model. *n* represents the total number of unfolding events, where the number in the bracket represents the total number of molecules of each sequence. (**E**) The time evolution of the folding probability *p*_fold_(t). Data represent the mean ± SD from three different DNA tethers. TB-1 (red), TB-8 (orange), TB-2 to TB-7 (cyan), and T30695 (gray). The TB-1 shows the fastest refolding among TB-1 to TB-8. (**F**) The steady-state folding probability *p*_st_. The red columns represent the fraction of fully-folded G4s (unfolding forces > 40 pN) and the gray columns represent the less stable states (unfolding forces < 40 pN). (**G**) Apparent folding rates *k*_fold_ of T30695 and TB-1 to TB-8.

We first analyzed G4s with a 1 nt thymine bulge between two adjacent guanine residues at 8 different positions (Table 1, TB-1 to TB-8). A previous NMR study (10) and CD spectra measurements showed that the TB-1 to TB-8 and T30695 sequences form parallel-stranded G4 structures in the presence of 100 mM KCl (Figure 1A). To characterize the mechanical stability, we used single-molecule magnetic tweezers (Figure 1B) to measure the unfolding force distribution based on a force-ramp procedure (Supplementary Figure S1), as described previously (40,41).

The unfolding force distributions of the TB-1 to TB-8 and T30695 sequences exhibit multiple unfolding force peaks, suggesting that these sequences form multiple conformations (Figure 1C-D). The TB-2 to TB-7 sequences show a major peak centered at ∼55 pN and a minor peak centered at ∼16 pN (Figure 1D, Supplementary Table S2), which are similar to the T30695. The average unfolding step sizes of the major form of TB-2 to TB-7 are consistent with the total number of nucleotides in fully-folded bulged G4s (Supplementary Figure S2A-B). This result suggests that the TB-2 to TB-7 sequences primarily form fully-folded parallel-stranded G4s with high mechanical stability. By fitting the unfolding force distributions with Bell's model (Supplementary, data analysis) (44,45), we obtained the zero-force unfolding rates *k*_unfold_ for the fully-folded bulged G4s (TB-2 to TB-7), which were in the range of 10^–5^ to 10^–7^ s^–1^ (Supplementary Figure S2C). Previously, we showed that T30695 and other canonical parallel-stranded G4s have a major unfolding force peak at 40–60 pN, which is associated with zero-force unfolding rates of 10^–5^ to 10^–7^ s^–1^ (37). This result suggests that the characteristics of high mechanical stability and slow unfolding rates can be applied to noncanonical G4s with a 1 nt bulge.

In contrast to TB-2 to TB-7 sequences, the TB-1 sequence, which has a bulge near the 5′ end, reveals large fractions of unfolding events at < 40 pN. By fitting to Bell's model, we obtained three unfolding force peaks for the TB-1 sequence (∼22 pN, ∼36 pN and ∼47 pN) and the fractions of each peak (62%, 25% and 7%). The average unfolding step sizes (16 nt) of the mechanically stable state is consistent with the total number of nucleotides of the fully-folded TB-1 G4 structure (Supplementary Figure S3A). The unfolding step sizes of the two less stable states were determined to be 14 nt and 13 nt, suggesting the formation of partially-folded G4s. When the time for refolding at 1 pN was reduced from more than 30 s to 1 s, the TB-1 sequence formed only two less stable states, suggesting that the less stable states were kinetically favored states (Supplementary Figure S3). The TB-8 sequence shows two unfolding force peaks centered at ∼31 pN (fraction = 33%) and ∼46 pN (fraction = 63%) with unfolding step sizes of 14 nt and 16 nt, respectively, indicating the formation of partially-folded and fully-folded G4s (Supplementary Figure S3).

We next measured the time-evolution folding probability *p*_fold_ (t) of TB-1 to TB-8 sequences. The DNA constructs were held at low force of 1 pN for various holding time intervals *t* and then unfolded in a force-ramp stretching cycle. The folded states reveal an unfolding event, while the unfolded or misfolded states reveal single-stranded DNA (ssDNA) like mechanical behavior in a subsequent stretching cycle. The time-evolution folding probability of each sequence was fitted with a single-exponential function, *p*_fold_ (t) = *p*_st_ [1 – exp(-*k*_fold_t)], where *p*_st_ is the steady-state folding probability and *k*_fold_ is the apparent folding rate of all folded species (Figure 1E). Based on the unfolding forces, we also estimated the *p*_st_ values of fully-folded G4s (unfolding forces > 40 pN, Figure 1F red column) and partially-folded G4s (unfolding forces < 40 pN, Figure 1F, gray column). The TB-1 and TB-8 sequences exhibit relatively large fractions of partially-folded G4s (87% and 33%) and high *p*_st_ (94% and 96%). In contrast, the TB-2 to TB-7 sequences primarily form fully-folded G4 and show *p*_st_ in the range of 58–79% (Figure 1F), suggesting that a 1 nt bulge at the middle increased the unfolded or misfolded fractions. The apparent folding rate of the TB-1 (*k*_fold_ = 0.11 ± 0.03 s^–1^) sequence was one order of magnitude higher than that of the TB-2 to TB-7 sequences (*k*_fold_ = ∼0.01-0.03 s^–1^) (Figure 1G), which also supports that the TB-1 sequence folds into kinetically preferred partially-folded G4s.

### Sequences with a bulge near the 5′ or 3′ end form intermediates with a guanine-vacancy

**Figure 2.**
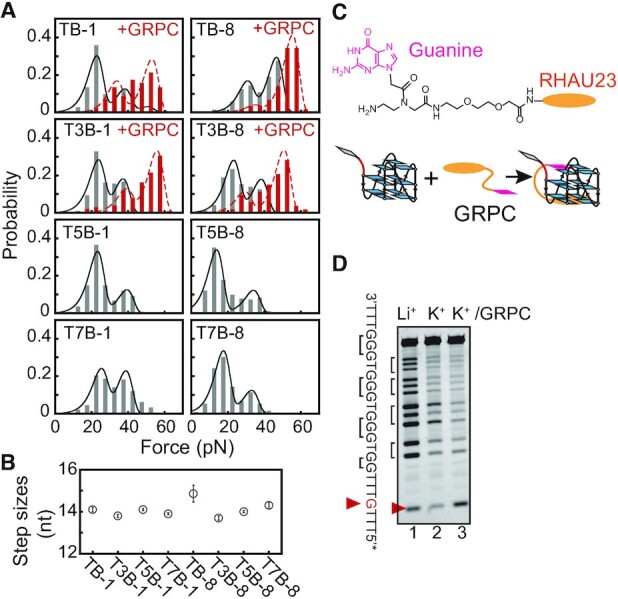
G4-forming sequences with a bulge near the 5′ or 3′ end mainly form GVBQs. (**A**) Unfolding force distributions of TB-1 to T7B-1 and TB-8 to T7B-8 (gray column). Stabilization of GVBQs by 0.5 μM GRPC (red columns). The total number of unfolding events and molecules are shown in Supplementary Table S3. (**B**) Average unfolding step sizes. (**C**) The guanine-RHAU23 peptide conjugate (GRPC) binds and stabilizes GVBQs. (**D**) DMS footprinting of the T3B-1 sequence in the presence of 0.5 μM GRPC.

To analyze the effects of bulge length, we measured the mechanical stability of G4-forming sequences bearing a different number of thymines between successive guanine pairs near the 5′ end (T3B-1, T5B-1, and T7B-1) and 3′ end (T3B-8, T5B-8 and T7B-8) (Table 1). As the bulge length increases, all the sequences form two less stable states with unfolding forces of ∼20 and ∼36 pN (Figure 2A, gray column, Supplementary Table S2). Notably, the unfolding step sizes of G4s with different lengths of a bulge near the 5′ end (TB-1, T3B-1, T5B-1 and T7B-1) and 3′ end (TB-8, T3B-8, T5B-8 and T7B-8) are all ∼14 nt, which means that they are shorter than the fully-folded G4 structures (Figure 2B, Supplementary Figure S4). The ∼14 nt unfolding step sizes are consistent with partially-folded G4 structures with a peeled guanine, suggesting that the guanine at the 5′ or 3′ end does not participate in the G-tetrad core (also called GVBQs). In addition, both the folding probability (*p*_st_ ∼90%, Supplementary Figure S5A and B) and folding rates (*k*_fold_ ∼0.1 s^–1^, Supplementary Figure S5C) of the TB-1 and TB-8 groups were independent of the bulge length, suggesting that these sequences with a bulge near their 5′ or 3′ ends were kinetically trapped in intermediate states.

To determine whether the intermediates of TB-1 and T3B-1 are GVBQs, we added GRPC to selectively target GVBQs (Figure 2C) (24). In the presence of a saturated concentration of 0.5 μM GRPC, TB-1 and T3B-1 show two unfolding force peaks at ∼33 pN, ∼53 pN and at ∼40 pN, ∼55 pN, respectively (Figure 2A, red column). The ∼20 pN major unfolding force peaks of TB-1 and T3B-1 disappear, and a new unfolding force peak at ∼54 pN appears, suggesting that the GRPC binds the intermediate of TB-1 and T3B-1 and increases the unfolding forces from ∼20 to ∼54 pN. The complementation of the peeled 5′ end guanine of the intermediates by GRPC results in similar mechanical stability as fully-folded parallel-stranded G4. Similar stabilization effects by GRPC were obtained for the TB-8 and T3B-8 G4s, suggesting that GRPC can also bind and stabilize 3′ end peeled GVBQs. It should be noted that there is more than one intermediate for TB-1 and T3B-1 and the ∼36 pN peak of TB-1 and T3B-1 is retained, suggesting that GRPC cannot stabilize this intermediate. The GRPC also reduced the folding probability *p*_fold_ probably due to the interactions between GRPC and other states (Supplementary Figure S5D).

To further confirm that GRPC stabilizes intermediate G4 through filling in guanine at the 5′ or 3′ end, we performed DMS footprinting experiments (Figure 2D). The 5′ guanine of T3B-1 shows stronger cleavage in the presence of 100 mM K^+^ and 0.5 μM GRPC (K^+^/GRPC) compared with 100 mM K^+^ only. This result suggests that the binding of GRPC to partially-folded T3B-1 blocks the conversion from partially-folded GVBQs to fully-folded G4s, thereby resulting in stronger cleavage of 5′ guanine. The TB-1 sequence does not exhibit stronger cleavage of 5′ guanine in the presence of K^+^/GRPC, probably because TB-1 has a faster rate than T3B-1 to fold into fully-folded G4s (Supplementary Figure S6). TB-8 and T3B-8 sequences also revealed stronger cleavage of the peeled 3′ guanine in the presence of K^+^/GRPC than in the presence of only K^+^ (Supplementary Figure S6). Taken together, these results indicate that G4-forming sequences with a bulge near the 5′ or 3′ end kinetically access partially-folded GVBQs, which can be selectively stabilized by GRPC.

### Mechanical stability of GVBQs depends on the positions of missing guanine

**Figure 3.**
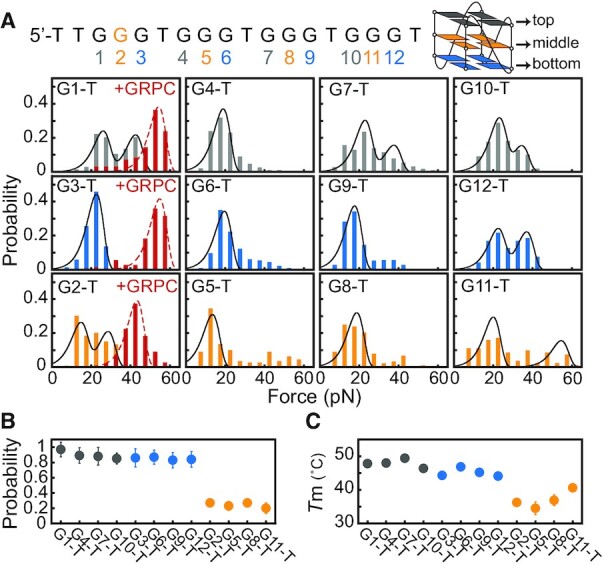
Mechanical stability of GVBQs. (**A**) Unfolding force distributions of the G1-T to G12-T. The G-to-T substitutions at the top, bottom, and middle tetrads are shown in gray, blue, and orange columns, respectively. Stabilization of GVBQs by 0.5 μM GRPC (red columns). (**B**) The steady-state folding probability *p*_st_. (**C**) Melting temperatures (*T*_m_s) of G1-T to G12-T DNA measured in 20 mM KCl.

To confirm that the GVBQs have weaker mechanical stability than fully-folded G4s, we also analyzed 12 G4-forming sequences with a guanine-to-thymine substitution (named G1-T to G12-T) (Supplementary Table S1). CD spectra (Supplementary Figure S7A) and a previous NMR study (13) suggested that these sequences form parallel-stranded GVBQs. The unfolding force distributions of 12 G-to-T substitution mutants measured using the same force-ramp procedure show that the major unfolding force peaks are < 40 pN (Figure 3A, Supplementary Table S2), suggesting that GVBQs have lower mechanical stability than fully-folded parallel-stranded G4s. The presence of multiple peaks for G1-T, G2-T, G7-T, G10-T, G11-T and G12-T suggests the formation of multiple structures with different mechanical stabilities. After 0.5 μM GRPC was added to the flow chamber, the major unfolding force peaks for G1-T, G2-T, and G3-T sequences were shifted from ∼25, ∼17 and ∼22 pN to ∼53, ∼42 and ∼54 pN, respectively (Figure 3A). These results suggest that the binding of GRPC to GVBQs stabilized it and resulted in mechanical stability as high as fully-folded parallel-stranded G4, which is consistent with the results obtained from TB-1 and T3B-1 bulged G4s.

An interesting finding in our single-molecule measurements is that the folding probabilities of G4s with a G-to-T substitution strongly depend on the positions of the missing guanine. Based on the positions of the missing guanine, we grouped the sequences as top-tetrad, middle-tetrad, and bottom-tetrad mutants that contain G-tract 5′TGG, 5′GTG and 5′GGT, respectively. Both top-tetrad and bottom-tetrad mutants show high folding probability and rapid folding rates (top-tetrad *p*_st_ ∼90%, *k*_fold_ ∼0.09 s^–1^; bottom-tetrad *p*_st_ ∼85%, *k*_fold_ ∼0.07 s^–1^) compared with the middle-tetrad mutants (*p*_st_ ∼24%, *k*_fold_ ∼0.02 s^–1^) (Figure 3B, Supplementary Figure S7B–D). Addition of 0.5 μM GRPC increased the folding probability *p*_fold_ of middle tetrad G2-T but reduced the *p*_fold_ of G1-T and G3-T though the reason remains unclear (Supplementary Figure S7E). The low folding probability of middle-tetrad mutants suggests that the abolished consecutive guanine pairs in one column of the G-tetrad core strongly hindered stable G4s formation. This finding is consistent with a previous single-molecule study which showed that a middle tetrad G-to-T mutant of telomeric G4s displayed unstructured ssDNA-like behavior in 100 mM K^+^ (35). The position-dependent folding probability is correlated with the thermodynamic stability (*T*_m_s) of G4s (Figure 3C). The top-tetrad and bottom-tetrad mutants show a considerably higher *T*_m_ (top-tetrad, *T*_m_ ∼48°C, bottom-tetrad *T*_m_ ∼44°C) than the middle-tetrad mutants (*T*_m_ ∼36°C) in 20 mM KCl (Figure 3C, Supplementary Figure S7F), suggesting that the middle-tetrad mutants have much lower thermodynamic stability. The lower melting temperature of guanine substitution in the middle-tetrad have also been observed for G-to-^oxo^G and G-to-A substitutions of the telomeric G4-forming sequence (46,47), suggesting that this is a general feature of guanine substitution mutants.

### Mechanical stability and bulge-length dependence of G4s with a ≥ 2 nt bulge in the middle

To evaluate the effects of bulge positions and bulge lengths in the middle of the sequences, we measured the mechanical stability of T2B-2 to T2B-7 sequences with a 2 nt bulge (Table 1). CD spectra show that all these sequences fold into parallel-stranded G4 structures (Supplementary Figure S8). Based on the positions of the bulge, we grouped the sequences with a bulge at position 2, 4, 6 (T2B-2, T2B-4, and T2B-6) as lower bulged G4s and the sequences with a bulge at position 3, 5, 7 (T2B-3, T2B-5, and T2B-7) as upper bulged G4s. The unfolding forces and unfolding step sizes suggest that the lower bulged G4s mainly form fully-folded G4s with high mechanical stability (Supplementary Figure S8). Comparing with G4s with a 1 nt lower bulge (Figure 1), the increased 2 nt bulge length reduced the folding probability (Supplementary Figure S8). In contrast to lower bulged G4s, the upper bulged G4s exhibit much broader unfolding force distributions between 5 and 60 pN which suggests the formation of multiple structures with distinct mechanical stability. The differences between upper and lower bulged G4-forming sequences could depend on the G4s topology.

**Figure 4.**
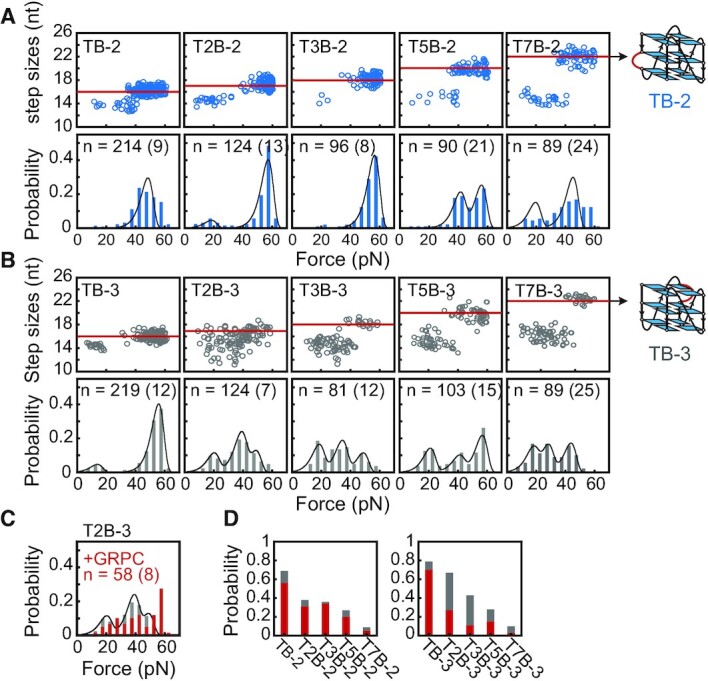
Mechanical diversity of G4s with a bulge in the middle. (**A, B**) Unfolding step sizes and unfolding force distributions of TB-2 to T7B-2 (A) and TB-3 to T7B-3 (B). The red lines and arrows present the total number of nucleotides in fully-folded G4s. (**C**) Unfolding force distribution of T2B-3 measured in the presence of 0.5 μM GRPC (red columns). (**D**) The steady-state folding probability *p*_st_. The data of T7B-2 and T7B-3 represent the folding probability measured at 300 s. The red columns represent the fraction of fully-folded G4s (unfolding forces > 40 pN), and the gray columns represent the less stable states (unfolding forces < 40 pN).

Next, we increased the length of thymines at position 2 (TxB-2: T2B-2, T3B-2, T5B-2, T7B-2) and position 3 (TxB-3: T2B-3, T3B-3, T5B-3, T7B-3) (Table 1). The T2B-2, T3B-2 sequences show a major peak at ∼56 pN (Figure 4A, Supplementary Table S2), while other sequences show broad unfolding force distributions with multiple peaks (Figure 4B). The structures with unfolding forces > 40 pN reveal unfolding step sizes of fully-folded G4s, while the structures with unfolding forces < 40 pN reveal smaller unfolding step sizes indicating the formation of partially-folded G4s (Figure 4A-B). To test whether the partially-folded structures contain GVBQ structures, we measured the unfolding forces of T2B-3 in the presence of 0.5 μM GRPC (Figure 4C). The < 40 pN species of T2B-3 were retained suggesting that these partially-folded structures cannot be stabilized by GRPC, which implies the formation of other conformations. By analyzing the time-dependent folding probability of TxB-2 and TxB-3 sequences, we obtained the steady-state folding probability *p*_st_ and folding rates *k*_fold_ (Supplementary Figure S9). Due to the low folding probability, the folding probability *p*_fold_(*t*) of the T7B-2 and T7B-3 measured after holding the DNA at low force for 300 s were used for comparisons (Figure 4D). The overall *p*_st_ of TxB-2 and TxB-3 decrease from 69% (TB-2) and 79% (TB-3) to 9% (T7B-2) and 10% (T7B-3), when the bulge length increases from 1 to 7 nt. For the TxB-3 group, the fraction of fully-folded G4s was lower than 15% when the bulge was > 2 nt (Figure 4D, red column). This result indicates that as the bulge became longer, the middle bulge significantly reduced the folding probability of fully-folded and stable partially-folded G4s.

## DISCUSSION

**Figure 5.**
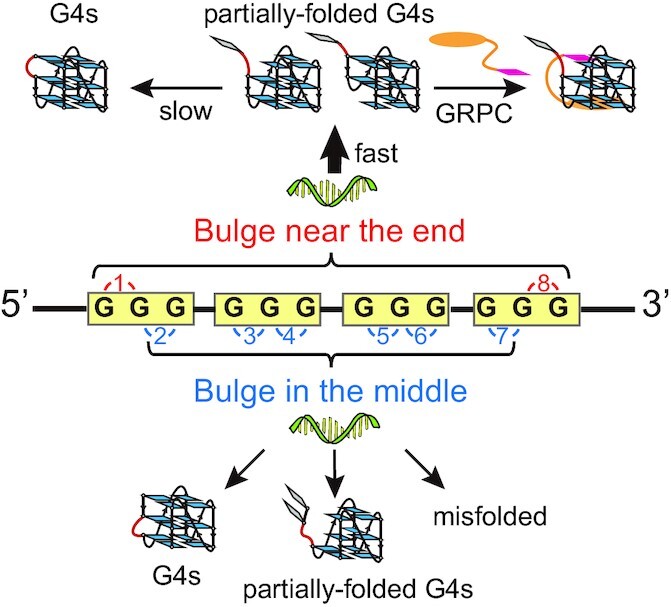
G4-forming sequences with a bulge can form multiple structures, including fully-folded G4s, partially-folded G4s, and misfolded. Sequences with a bulge near the 5′ and 3′ end mainly form partially-folded intermediates and slowly convert to fully-folded G4s (upper panel). The major intermediates are GVBQs which can be selectively stabilized by GRPC. A ≥2 nt middle bulge significantly reduced the folding probability and increase the unfolded or misfolded fractions (lower panel).

Mechanical diversity and partially-folded G4s such as triplexes have been well documented for canonical G4s (29,30,35,37,48). However, the polymorphism and the effects of a bulge on the mechanical stability of noncanonical G4s have not been investigated before. We systematically analyzed the mechanical stability of bulged G4s and our results suggest that G4-forming sequences with a bulge can form multiple structures, including fully-folded G4s (unfolding forces > 40 pN), partially-folded intermediates (unfolding forces < 40 pN) (Figure 5). This result is in contrast to the findings of previous NMR studies, which revealed that only fully-folded TB-1 G4s with all 12 guanines participate in the formation of the G-tetrad core (9,10). This discrepancy may be observed because the G4s prepared in NMR measurements undergo thermal annealing processes over several hours, while the G4s refold in the force-ramp procedure is a seconds-to-minutes time-scale. Many studies have revealed that reaching the equilibrium state (thermodynamic minima) of G4s requires a long time period (up to days) (49). The kinetically preferred intermediates may be biologically relevant states, as many biologically relevant processes, for example, transcription, occur on a seconds-to-minutes time scale. The diverse intermediates with different mechanical stability may function as different barriers to the progression of motor proteins, for example, helicases or polymerases (38).

The identification of GVBQs as major intermediates of end bulged G4s is important for understanding the folding pathways of bulged G4s. The formation of GVBQs or strand-slipped G4s as a folding intermediate has been proposed through molecular dynamics simulations (50,51). However, there was no direct evidence that G4s can form via GVBQ intermediates. Based on the unfolding step sizes and unfolding forces, we established for the first time that GVBQs are major intermediates of G4s with a bulge near the 5′ or 3′ end while the 5′ or 3′ end guanine flips out and does not participate in the formation of G4. With increasing time, the GVBQs slowly convert to fully-folded bulged G4s (Supplementary Figure S3). Figure 5 upper panel shows two potent GVBQs structures while the first GVBQs can transition to the fully-folded G4s by simply docking of the 5′ guanine and the second GVBQs must undergo strand shift before the 5′ guanine can dock into the structure. The strand shift may take a long time thus explaining the slow conversions from the GVBQs to fully-folded G4s. Our data show extreme mechanical diversity of middle bulged G4s (T2B-3), suggesting the formation of more than one partially-folded intermediates. We also provide a new strategy to modulate G4s functions by stabilizing kinetically preferred GVBQ intermediates. By using GRPC specifically to stabilize GVBQs, we demonstrated that selective filling in of the guanine of GRPC into the G-vacancy can achieve high mechanical stability as fully-folded G4s, thus represent promising targets for drug design.

The systematic analysis of the effects of bulge lengths and positions on the mechanical stability of G4s may help to establish rules to predict the mechanical stability and folding probability of noncanonical G4s. Our previous measurements on canonical parallel-stranded G4s suggest that high mechanical stability is prevalent in fully-folded parallel-stranded G4s (37,40,52). The current study further demonstrates that parallel-stranded G4s with a bulge (fully-folded states) also exhibit high mechanical stability (unfolding forces > 40 pN), while parallel-stranded GVBQs exhibit low mechanical stability. In addition to previous reported melting temperatures (10), we show that folding probability, apparent folding rates, and folded populations depend on both bulge positions and bulge lengths (Figure 5). A bulge reduced the thermal stability of G4s probably because the bulge length dependent entropy cost of closing a loop or bulge in G4 structures (53,54). When a bulge is located near the 5′ or 3′ end, the sequences primarily form kinetically preferred GVBQs. The entropy cost for closing the bulge at the 5′ or 3′ end prevents the convention of GVBQs to fully-folded G4s. When a bulge is located in the middle of the sequences, a ≥ 2 nt middle bulge was observed to significantly reduce the folding probability and increase the unfolded or misfolded fractions. This is probably because both the fully-folded and intermediate states of middle bulged G4s would be expected to destabilzed by bulge-closure entropies. It should be noted that the unfolded or misfolded fractions may include mechanically weak conformations (such as two-quartet G4s) which could unfold at low force (< 4 pN) as reported by previous fluorescence-force spectroscopy measurements (35). At low forces, the end-to-end extension of ssDNA (∼16 nt) and misfolded G4s are similar (unfolding step sizes < 3 nm), thus cannot be directly detected by force-based measurements (43). The mechanically weak conformations hindered the formation of mechanically stable G4 structures, thereby reducing the folding probability, folding rate, and thermal stability of G4s.

## Supplementary Material

gkab531_Supplemental_File
