# Supplementary material for: Mechanical diversity and folding intermediates of parallel-stranded G-quadruplexes with a bulge
Source: Nucleic Acids Res. 2021 Jun 17;49(12):7179–88. doi: 10.1093/nar/gkab531 (PMC8266575; doi:10.1093/nar/gkab531)
Supplement: gkab531_Supplemental_File [file gkab531_supplemental_file.pdf]

Mechanical diversity and folding intermediates of parallel-stranded G-quadruplexes with a bulge

Yashuo Zhang[1], Yuanlei Cheng[1], Juannan Chen[2], Kewei Zheng[2,*], and Huijuan You[1,*]

[1]Hubei Key Laboratory of Natural Medicinal Chemistry and Resource Evaluation, School of Pharmacy, Tongji Medical College, Huazhong University of Science and Technology, Wuhan, 430030, China

[2]School of Pharmaceutical Sciences (Shenzhen), Sun Yat-Sen University, Guangzhou, 510275, China

*To whom correspondence should be addressed. Tel: 86-27-83692868; Fax: 86-27-83692762; Email: youhuijuan@hust.edu.cn. Correspondence may also be addressed to zhengkw6@mail.sysu.edu.cn.

The authors wish it to be known that, in their opinion, the first 2 authors should be regarded as joint First Authors.

Table of Contents

Data analysis

Table S1-S3

Figure S1-S9

Data analysis

Fitting the unfolding force distribution. The unfolding force distribution $p(f)$ was analyzed by Bell's model. It fits an unfolding force distribution with a single force peak as

$$p(f) = \frac{k_u^0}{\gamma} \exp\left\{\frac{\Delta x f}{k_\mathrm{B} T} + \frac{k_B T k_u^0}{\Delta x \gamma}\left[1 - \exp\left(\frac{F \Delta x}{k_B T}\right)\right]\right\}$$

,where k_u^0 is zero force unfolding rate, k_B is Boltzmann constant, T is absolute temperature, r is the loading rate，Δx_u is the transition distance. For sequences show two or three unfolding force peaks, the combinations of two or three unfolding species were used $p(f) = \alpha_1 p_1^{Bell}(f) + (1 - \alpha_1) p_2^{Bell}(f)$ or $p(f) = \alpha_1 p_1^{Bell}(f) + \alpha_2 p_2^{Bell}(f) + (1 - \alpha_1 - \alpha_2) p_3^{Bell}(f)$, where α_n are the ratio of each species. The k_u^0 and α_n of each species were treated as fitting parameters. We used fixed Δx_u to analyze all species and Δx_u = 1.0 nm was the best fitting parameter obtained by Matlab from analyzing T30695 and TB-1 to TB-8. The correlation coefficient of one and multiple peaks fitting were used to find the best fitting. The center of peaks was identified by Gaussian fitting.

Table S1. GVBQ-forming sequences used in this study

Name	Sequence (5′ to 3′)			
G1-T	TTTGGT	GGGT	GGGT	GGGT
G2-T	TTGTGT	GGGT	GGGT	GGGT
G3-T	TTGGTT	GGGT	GGGT	GGGT
G4-T	TTGGGT	TGGT	GGGT	GGGT
G5-T	TTGGGT	GTGT	GGGT	GGGT
G6-T	TTGGGT	GGTT	GGGT	GGGT
G7-T	TTGGGT	GGGT	TGGT	GGGT
G8-T	TTGGGT	GGGT	GTGT	GGGT
G9-T	TTGGGT	GGGT	GGTT	GGGT
G10-T	TTGGGT	GGGT	GGGT	TGGT
G11-T	TTGGGT	GGGT	GGGT	GTGT
G12-T	TTGGGT	GGGT	GGGT	GGTT

Table S2. The mechanical stability of G4s analyzed in this study

Name	Unfolding force (pN)	$p_{fraction}$	p_{st}	k_{fold} (s^{-1})	k_{unfold} (s^{-1})
T30695	22±3; 56±3	0.20:0.67	0.87±0.07	0.20±0.07	$(2±1)×10^{-3}$; $(5.2±0.8)×10^{-7}$
TB-1	22±3; 36±4; 47±7	0.62:0.25:0.07	0.94±0.07	0.11±0.03	$(2.0±0.3)×10^{-3}$; $(5±2)×10^{-5}$; $(2±4)×10^{-6}$
TB-2	47±6	—	0.69±0.04	0.028±0.005	$(3.7±0.9)×10^{-6}$
TB-3	13±4; 55±4	0.08:0.71	0.79±0.10	0.014±0.006	$(1.7±0.3)×10^{-2}$; $(6.1±0.2)×10^{-7}$
TB-4	16±5; 56±3	0.06:0.52	0.58±0.04	0.018±0.003	$(9±7)×10^{-3}$; $(5.3±0.5)×10^{-7}$
TB-5	19±6; 57±4	0.06:0.53	0.59±0.07	0.017±0.006	$(5±3)×10^{-3}$; $(3.6±0.2)×10^{-7}$
TB-6	18±5; 55±5	0.17:0.47	0.64±0.05	0.023±0.006	$(5.1±0.6)×10^{-3}$; $(6.3±0.3)×10^{-7}$
TB-7	16±5; 57±3	0.29:0.36	0.65±0.15	0.015±0.007	$(6±1)×10^{-3}$; $(4.2±0.6)×10^{-7}$
TB-8	31±6; 46±4	0.33:0.63	0.96±0.11	0.022±0.007	$(2.9±0.3)×10^{-4}$; $(5.7±0.3)×10^{-6}$
T3B-1	23±3; 36±5	0.56:0.39	0.95±0.05	0.11±0.02	$(1.6±0.3)×10^{-3}$; $(6±2)×10^{-5}$
T5B-1	22±4; 39±4	0.64:0.25	0.89±0.11	0.08±0.03	$(1.7±0.1)×10^{-3}$; $(3.4±0.7)×10^{-5}$
T7B-1	24±4; 39±6	0.45:0.47	0.92±0.10	0.15±0.09	$(1.1±0.2)×10^{-3}$; $(3.9±0.7)×10^{-5}$
T3B-8	21±4; 35±7	0.53:0.36	0.89±0.11	0.10±0.06	$(1.8±0.3)×10^{-3}$; $(4±1)×10^{-5}$
T5B-8	14±4; 33±6	0.60:0.22	0.82±0.09	0.13±0.04	$(1.7±0.2)×10^{-2}$; $(1.2±0.5)×10^{-4}$
T7B-8	16±5; 33±4	0.51:0.20	0.71±0.07	0.10±0.05	$(6.9±0.6)×10^{-3}$; $(1.6±0.3)×10^{-4}$
T2B-2	17±6; 57±3	0.04:0.34	0.38±0.03	0.02±0.02	$(6±5)×10^{-3}$; $(4.3±0.5)×10^{-7}$
T3B-2	55±4	—	0.36±0.02	0.010±0.002	$(5.2±0.2)×10^{-7}$
T5B-2	41±5; 56±3	0.12:0.15	0.27±0.03	0.005±0.002	$(2.2±0.3)×10^{-5}$; $(6.1±0.7)×10^{-7}$
T7B-2	20±5; 47±6	0.02:0.06*	N.D.	N.D.	$(4±2)×10^{-3}$; $(8±3)×10^{-6}$
T2B-3	19±4; 38±6; 55±3	0.1 6:0.32:0.19	0.67±0.13	0.015±0.006	$(4±1)×10^{-3}$; $(4.0±0.6)×10^{-5}$; $(3.4±0.9)×10^{-6}$
T3B-3	18±3; 34±7; 50±2	0.15:0.17:0.11	0.43±0.03	0.0040±0.0006	$(6±1)×10^{-3}$; $(1.2±0.3)×10^{-4}$; $(3±1)×10^{-6}$
T5B-3	20±5; 40±6; 56±3	0.09:0.05:0.14	0.28±0.06	0.004±0.002	$(2.9±0.8)×10^{-3}$; $(2±1)×10^{-5}$; $(4.9±0.9)×10^{-7}$
T7B-3	19±6; 29±3; 43±5	0.03:0.03:0.04*	N.D.	N.D.	$(7±1)×10^{-3}$; $(5.1±0.8)×10^{-4}$; $(1.1±0.1)×10^{-5}$
T2B-4	20±6; 57±4	0.03:0.27	0.30±0.04	0.016±0.007	$(4±4)×10^{-3}$; $(4.4±0.7)×10^{-7}$
T2B-5	17±6; 49±6	0.20:0.57	0.77±0.20	0.019±0.009	$(6±2)×10^{-3}$; $(2.7±0.4)×10^{-6}$
T2B-6	20±7; 56±4	0.04:0.33	0.37±0.05	0.018±0.007	$(5±4)×10^{-3}$; $(4.6±0.6)×10^{-7}$
T2B-7	14±3; 27±5; 47±6	0.23:0.18:0.35	0.76±0.20	0.014±0.008	$(9±4)×10^{-3}$; $(5±2)×10^{-4}$; $(6±2)×10^{-6}$
G1-T	25±4; 41±6	0.49:0.48	0.97±0.09	0.10±0.04	$(9±2)×10^{-4}$; $(1.6±0.3)×10^{-5}$
G2-T	17±6; 30±3	0.14:0.13	0.27±0.01	0.02±0.01	$(1.4±0.4)×10^{-2}$; $(5±2)×10^{-4}$
G3-T	22±4	—	0.86±0.12	0.06±0.03	$(2.0±0.1)×10^{-3}$
G4-T	18±5	—	0.89±0.10	0.10±0.05	$(4.9±0.6)×10^{-3}$
G5-T	13±3	—	0.23±0.06	0.01±0.02	$(1.8±0.4)×10^{-2}$
G6-T	19±5	—	0.87±0.09	0.10±0.05	$(4.2±0.8)×10^{-3}$
G7-T	22±7; 37±4	0.57:0.31	0.88±0.12	0.10±0.07	$(1.9±0.4)×10^{-3}$; $(5±2)×10^{-5}$
G8-T	17±7	—	0.27±0.02	0.02±0.01	$(5±1)×10^{-3}$
G9-T	16±5	—	0.83±0.10	0.08±0.05	$(7±1)×10^{-3}$
G10-T	23±6; 37±3	0.55:0.30	0.85±0.07	0.06±0.02	$(1.9±0.2)×10^{-3}$; $(1.1±0.2)×10^{-4}$
G11-T	21±6; 49±7	0.13:0.07	0.20±0.07	0.01±0.01	$(4±1)×10^{-3}$; $(8±4)×10^{-7}$
G12-T	21±5; 36±5	0.42:0.42	0.84±0.10	0.020±0.008	$(2.2±0.3)×10^{-3}$; $(6.0±0.9)×10^{-5}$

The center of unfolding force peak were obtained by fitting the unfolding force distribution to

Gaussian function. The fraction p_{fraction} ($p_{\text{fraction}} = \alpha_i p_{st}$) and k_{unfold} of each species were obtained by fitting unfolding force distributions to Bell's model. The p_{st} is the steady-state folding probability and k_{fold} is the apparent folding rates, which were obtained by fitting the time-evolution folding probability with a single-exponential function. *The fraction p_{fraction} of T7B-2 and T7B-3 were obtained at 300 s.

Table S3. The total number of unfolding events (n) and the total number of molecules (in the bracket) of following DNA.

Sequence	n	Sequence	n	Sequence	n
TB-1	245 (8)	G1-T	206 (6)	G1-T+GRPC	96 (10)
T3B-1	192 (10)	G2-T	119 (23)	G2-T+GRPC	142 (13)
T5B-1	249 (7)	G3-T	218 (10)	G3-T+GRPC	142 (9)
T7B-1	241 (11)	G4-T	188 (9)		
TB-8	257 (19)	G5-T	110 (19)		
T3B-8	222 (7)	G6-T	308 (14)		
T5B-8	191 (8)	G7-T	216 (15)		
T7B-8	132 (6)	G8-T	88 (16)		
TB-1+GRPC	126 (8)	G9-T	280 (14)		
T3B-1+GRPC	98 (14)	G10-T	208 (7)		
TB-8+GRPC	58 (9)	G11-T	81 (26)		
T3B-8+GRPC	53 (15)	G12-T	354 (18)		

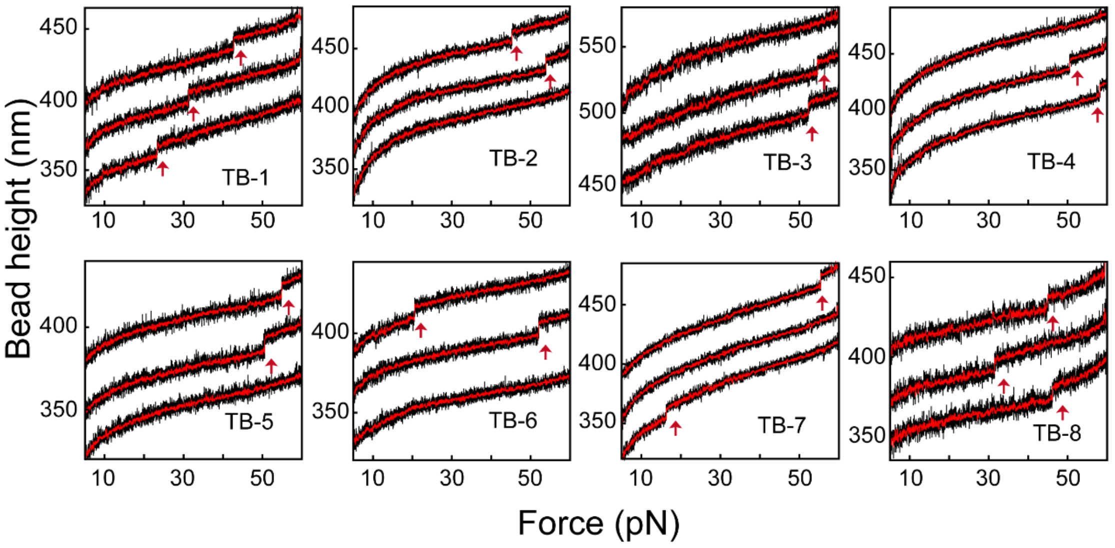

Figure S1. Representative force-bead height curves of TB-1 to TB-8 DNA measured in force-ramp experiments. Raw data (black), smoothed data (red). For visual clarity the three stretching curves are shifted 30 nm along the extension axis. The G4 unfolding causes a sudden extension increases (red arrows).

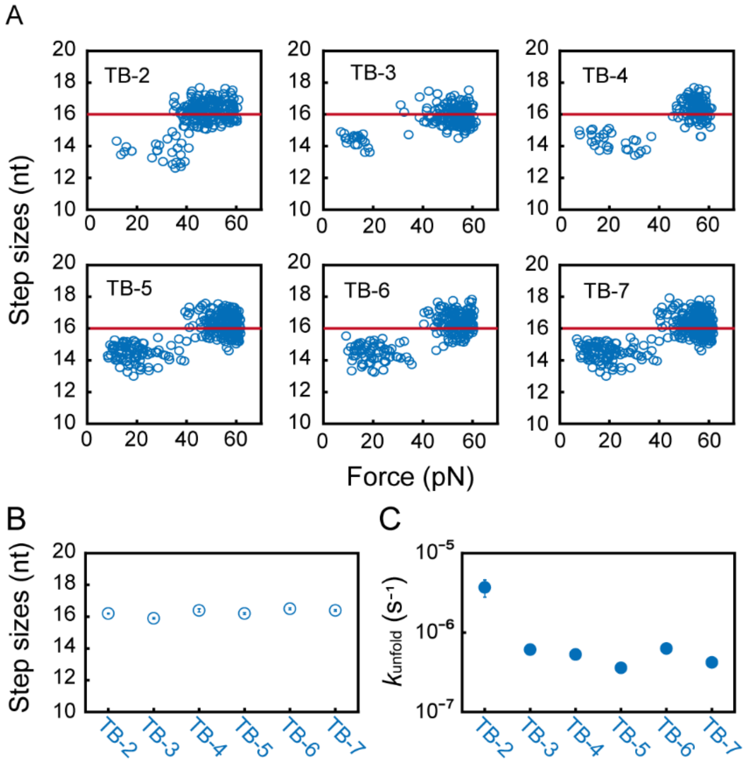

Figure S2. G4s bearing a 1 nt bulge in the middle. (A) Scatter plots of unfolding step sizes of TB-2 to TB-7 sequences. The red lines present the total number of nucleotides in fully-folded G4s (16nt). (B) Average unfolding step sizes of major form of TB-2 to TB-7. (C) Unfolding rates of fully-folded TB-2 to TB-7 G4s.

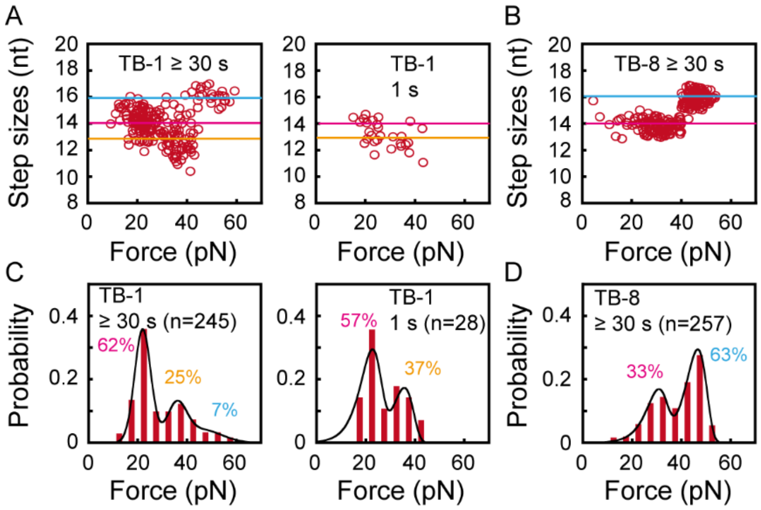

Figure S3. G4s bearing a 1 nt bulge near the 5' and 3'-end. (A-B) Scatter plots of unfolding step sizes and unfolding forces of TB-1 (A) and TB-8 (B). TB-1 was hold at low force for 1 s (left) and ≥ 30 s (right) for refolding (A). The lines present the average step sizes of stable state (blue) and two less stable states (pink and orange), respectively. (C-D) Unfolding force distributions. Data were fitted by Bell's model. *n* represents the number of stretching events. The blue, pink and orange numbers represent the fraction of each peak.

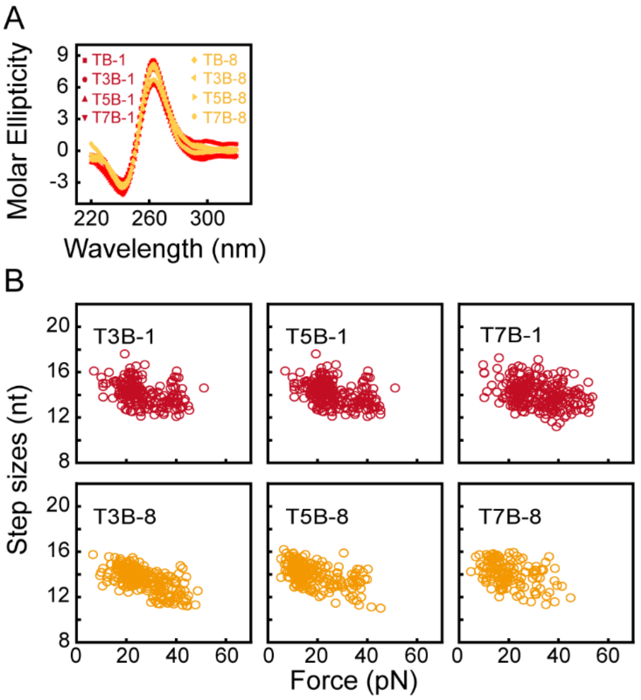

Figure S4. G4s bearing a 3 to 7 nt bulge near the 5' and 3' end. (A) CD spectra of TxB-1 and TxB-8. (B) Scatter plots of unfolding step sizes and unfolding forces of TxB-1 and TxB-8.

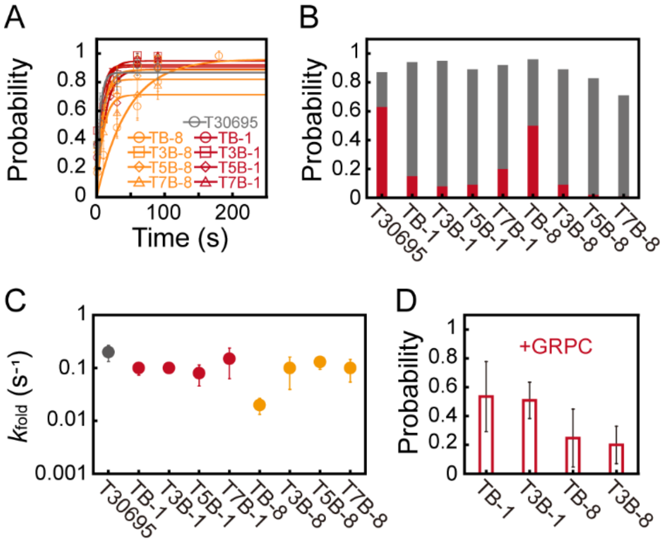

Figure S5. (A) p_{fold}(t) of T30695, TxB-1 and TxB-8. (B) Steady-state folding probability p_{st}. The red columns represent the fully-folded G4s and the gray columns represent the less stable states. (C) Folding rates. (D) 0.5 μM GRPC reduced the folding probability of TxB-1 and TxB-8. p_{fold} for TxB-1 and TxB-8 were measured at refolding time of 90 s and 300 s, respectively.

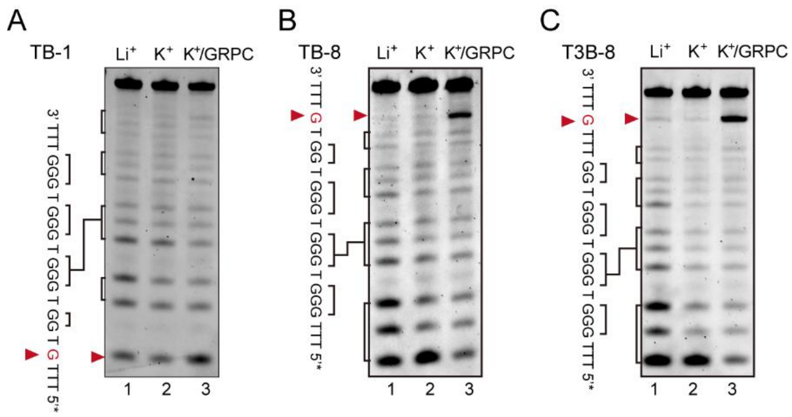

Figure S6. DMS footprinting of TB-1 (A), TB-8 (B), and T3B-8 (C). The red arrow represents the 5'-end guanine of TB-1 and 3'-end guanine of TB-8 and T3B-8. The bands show stronger cleavage in the presence of 100 mM K^+ and 0.5 μM GRPC than 100 mM K^+ only.

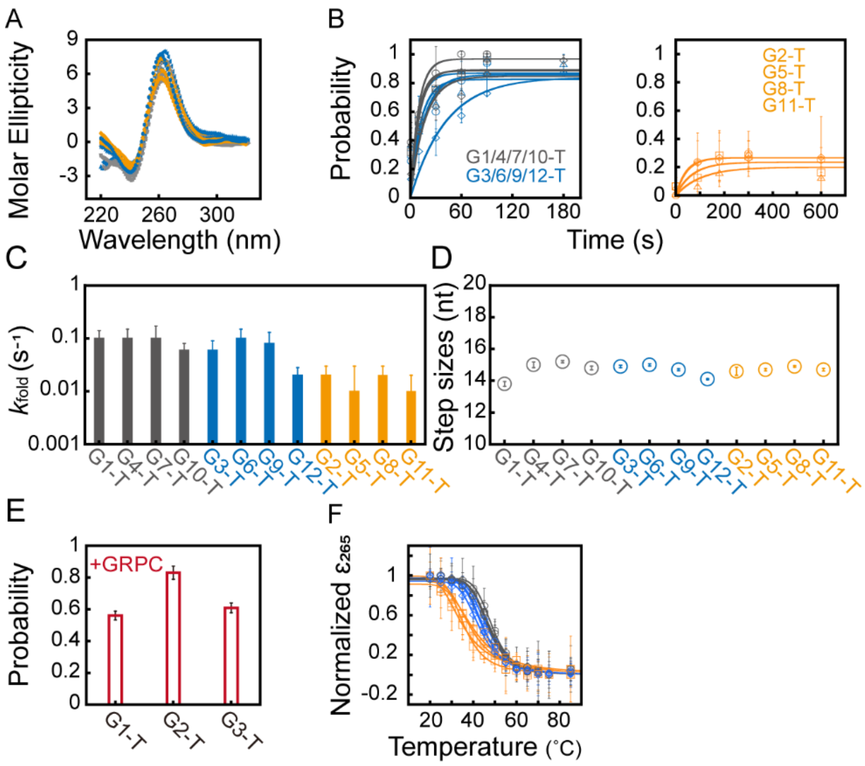

Figure S7. (A) CD spectra of 12 GVBQs. (B) The p_{st} of the 12 GVBQs. The top-tetrad, middle-tetrad, and bottom-tetrad mutants are shown in gray, orange, and blue, respectively. (C) Folding rates. (D) Average unfolding step sizes. (E) p_{fold} of G1-T, G2-T and G3-T measured in the presence of 0.5 μM GRPC at refolding time of 90 s. (F) Melting curves. The top-tetrad, middle-tetrad, bottom-tetrad mutants are shown in gray, orange, and blue, respectively. Low K^+

concentration (20 mM) was used to reduce the melting temperatures to experimentally accessible values.

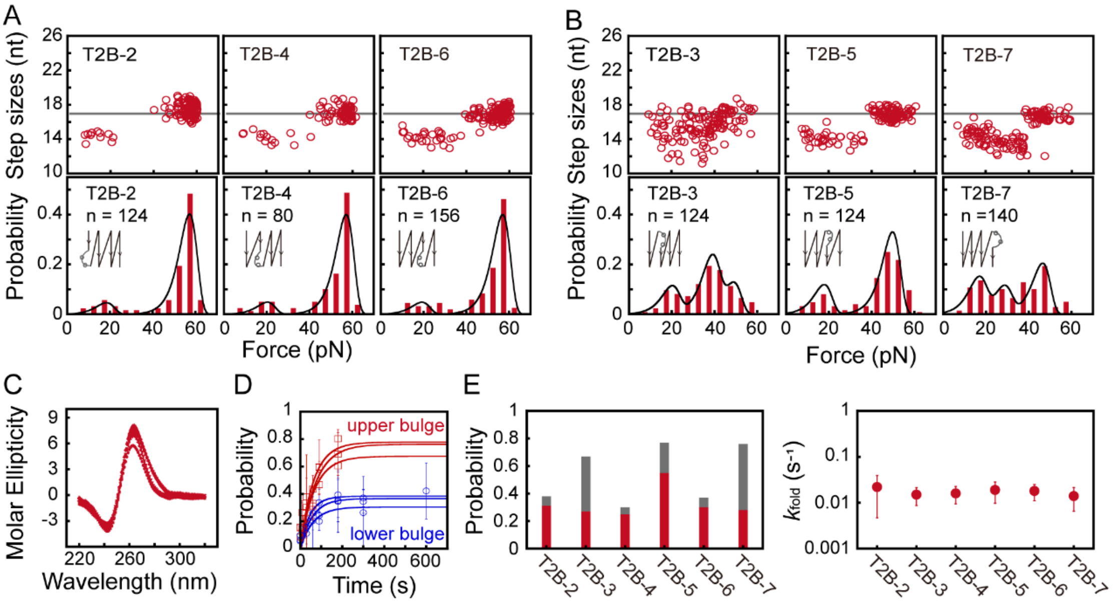

Figure S8. G4s bearing a 2 nt bulge in the middle. (A-B) Unfolding step sizes and unfolding forces. Upper bulged G4s: T2B-2, T2B-4, and T2B-6 (A), lower bulged G4s: T2B-3, T2B-5 and T2B-7 (B). The gray lines present the total number of nucleotides in fully-folded G4s (17 nt). Unfolding force histograms were fitted to Bell's model. (C) CD spectra. (D) $p_{fold}(t)$. Upper bulged G4s (red), lower bulged G4s (blue). (E) The p_{st} (left) and k_{fold} (right) of T2B-2 to T2B-7. The red columns represent the fraction of fully-folded G4s and the gray columns represent the fraction of less stable states.

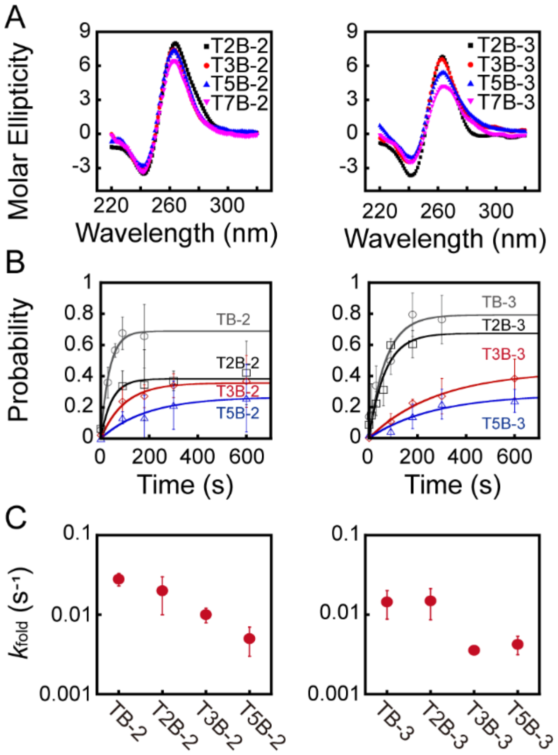

Figure S9. (A) CD spectra of TxB-2 (left) and TxB-3 (right) groups. (B) $p_{fold}(t)$ of TxB-2 (left) and TxB-3 (right) groups. Data represent mean ± SD from three different DNA tethers and were fitted by an exponential function. (C) Folding rates of TxB-2 (left) and TxB-3 (right) groups.
